# The Australian mixed-mode observing program

**DOI:** 10.1007/s00190-022-01657-2

**Published:** 2022-09-27

**Authors:** Lucia McCallum, Lim Chin Chuan, Hana Krásná, Jamie McCallum, Johannes Böhm, Tiege McCarthy, Jakob Gruber, Matthias Schartner, Jonathan Quick, Axl Rogers

**Affiliations:** 1 University of Tasmania, Private Bag 37, Hobart, 7001 Australia; 2 Technische Universität Wien, Vienna, Austria; 3 ETH Zürich, Zürich, Switzerland; 4 Hartebeesthoek Radio Astronomy Observatory, Krugersdorp, South Africa; 5 Auckland University of Technology, Auckland, New Zealand

**Keywords:** Very long baseline interferometry (VLBI), Mixed-mode observations, Terrestrial reference frame (TRF), AUSTRAL

## Abstract

Global geodetic VLBI is upgrading to its next-generation observing system, VGOS. This upgrade has turned out to be a process over multiple years, until VGOS reaches its full capabilities with the envisaged continuous observations. Until then, for the Australian stations, the upgrade means ceasing their legacy S/X observations, leaving a large gap in the global network as well as in the station time series. The Australian mixed-mode observing program is a series of sessions where the VGOS stations in Hobart and Katherine observe legacy S/X VLBI together with other stations in the region. This paper describes the technical details of these observations and their processing strategies and discusses their suitability for geodetic results by comparison with those of standard legacy S/X sessions. The presented mixed-mode sessions allow a continuation of the station time series, a benefit for the stations themselves as well as for future realisations of the terrestrial and celestial reference frames. A novel mode of observing is introduced and tested. The results are promising and it is suggested for acceptance into standard legacy S/X IVS observations, overcoming current gaps in the network due to VGOS upgrades and preventing a worsening of global results otherwise.

## Introduction

Global geodetic very long baseline interferometry (VLBI) operations are experiencing a transition to the new generation system, the VLBI Global Observing System (VGOS, Petrachenko et al. 2009; Niell et al. 2006). Currently, the bulk of the observations organised through the International VLBI Service for Geodesy and Astrometry (IVS, Nothnagel et al. 2017) are still in the dual-frequency *legacy* S/X mode, while the new system is gradually working towards its full operational capabilities (e.g. Niell et al. 2018).

Despite the fact that one of the initial aims for VGOS was to have a more uniform system, today it is clear that the actual VGOS realisations at the various geodetic observatories around the globe differ significantly (e.g. in frequency coverage, sampler bandwidth, or recorder logistics). With the currently used VGOS mode being based on limitations which do not necessarily apply to the whole network, greater utilisation of the actual resources and capabilities of the stations is increasingly sought after. While not dealing with VGOS observing per se, this work suggests one way for increased usage of the available infrastructure for improved results: namely by allowing stations which are currently underutilised to contribute to VLBI results. VGOS data volumes are huge, which, together with a lack of automation, currently restricts routine VGOS operations to a single 24-h session per week. With resources only slowly increasing, it is expected that it will be several years until VGOS will reach its goal of continuous observations. Until then, the legacy S/X observations will remain crucial in contributing to the IVS products and it is important to maintain best-possible VLBI products from S/X observations.

Australia’s contribution to the IVS is managed by the University of Tasmania (UTAS), funded through the federal body Geoscience Australia. Historical data shows a significant lack of observations from the Australian continent, with only the Hobart 26m telescope (Ho) or occasionally the Parkes observatory contributing to regular IVS observing (Titov 2007). The advent of the AuScope VLBI array (Lovell et al. 2013) in the year 2010 changed this dramatically, and the new telescopes in Hobart (Hb), Katherine (Ke) and Yarragadee (Yg) were soon amongst the busiest IVS stations in the network. Their observations also made an impact on global results: the achieved precision of regular observing programs for southern baselines finally matching those of the northern baselines (Plank et al. 2015b). As emphasised in the latest realisation of the international celestial reference system (ICRS), ICRF3 (Charlot et al. 2020), results for the southern hemisphere still clearly lag behind those of the northern sky, though regular as well as special observing campaigns (e.g. Plank et al. 2017; McCallum et al. 2017) from the AuScope array do have a positive impact. The intensive observing program with the AuScope VLBI network was interrupted, due to the upgrade to VGOS-compatible systems. The AuScope VLBI array was designed and built with VGOS in mind, with the S/X system installed as an interim solution, until VGOS receivers and other required technology were enabled. The antenna structures are slow VGOS antennas, with slew speeds of up to $5^{\circ }/s$ in azimuth and $\approx 1.25^{\circ }/s$ in elevation. The important aspect to note is that for the VGOS upgrade the same antennas are used with only the receivers and backends being upgraded. As a consequence, the telescopes become non-operational during the upgrade and lose their ability to observe legacy S/X VLBI in the traditional way.

Hb has not been observing the IVS VLBI programs since mid-2017 (as well as during two VGOS test periods in 2015 and 2016) and Ke left the legacy S/X observing program in mid-2019. Both stations are now in a VGOS configuration; however, they have not joined the IVS VGOS sessions beyond a fringe test. The main reason is that the current backend cannot support a full 4-band VGOS system. A solution is in production, with an upgraded DBBC3 (Digital Base Band Converter)^1^ sampler system recently installed and under testing at Hobart.

Nevertheless, the VGOS upgrade only leaves Yg (plus the occasional contribution of Ho)^2^ in the Australian legacy network, leaving a huge gap in the global network. Not being able to contribute to global observations is a problem for the AuScope VLBI project and a decision has been made to not upgrade Yg until routine observations at Hb and Ke can be re-established.

The current situation further leaves the AuScope array in an heterogeneous state, bringing the rather successful AUSTRAL observing program (Plank et al. 2017) to a halt. The AUSTRAL sessions have been organised with other closely collaborating institutions and southern hemisphere stations, with the aim of exploiting the full potential of the telescopes, increasing the cadence of the observations as well as building expertise for the full process chain from scheduling through to geodetic results. It is about making best possible use of the AuScope array, with the practical experience enabling good feedback and improvement in the systems. The need for observations in the VGOS test phase is clear, whether it is about initial feedback of system performance or the continuity of the station times series. Finally, the requirements in terms of data transport logistics, storage and automation for full VGOS operation are huge, so performing additional observations at high data rates will increase the preparedness of Australian stations in these matters as well.

**Fig. 1:**
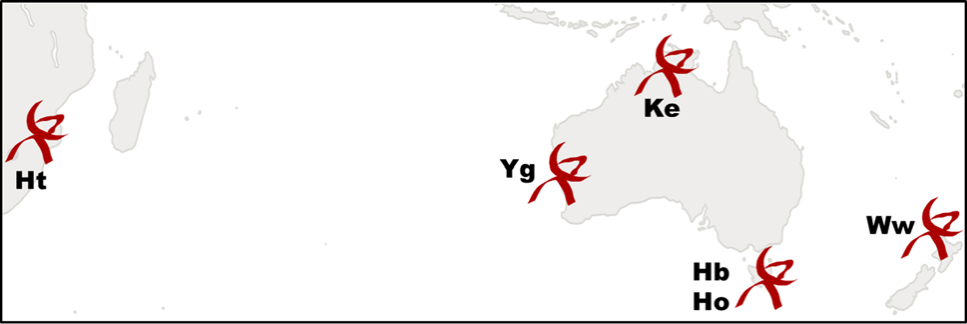
Network of the Australian mixed-mode sessions. The AuScope VLBI array consisting of Hobart 12m (Hb, VGOS), Katherine 12m (Ke, legacy in 2018, VGOS since mid-2019), Yarragadee 12m (Yg, legacy), and the Hobart 26m telescope (Ho, legacy) operated by the University of Tasmania. In some sessions the HartRAO15m (Ht, legacy) and the Warkworth 12m (Ww, legacy) telescopes join

As a response to the current status, the Australian mixed-mode program was started. All sessions with the IVS three-letter code AUM as well as AUA060 and higher are part of this program. Following the AUSTRAL program, these are observations on the regional Australian network with occasional inclusion of the telescopes at the Hartebeesthoek Radio Astronomical Observatory in South Africa (Ht) and in Warkworth, New Zealand (Ww) (Figure 1). These sessions are *mixed-mode*, in that the legacy S/X configuration is observed by the upgraded VGOS stations in different polarisations and with significantly different hardware. These mixed-mode sessions represent a rather new type of observations, with multiple modifications necessary in the observing setup (Sect. 2) as well as in post-processing (Sect. 4). Since 2018, a large set of sessions was successfully observed and processed (Sect. 3), revealing promising results (Sect. 5). As argued in the Conclusions (Sect. 6), this newly presented technique of mixed-mode observations seems to be viable and well-suited to overcome the current gap of the Australian AuScope stations in IVS, until hopefully the VGOS system will take over the routine observing program. The aim of this paper is twofold: firstly, it shall thoroughly describe these mixed-mode sessions and test whether the different nature of these observations cause any systematics in the results. After all, some of the described sessions (session codes AUA) will be part of the solution for the next release of the international terrestrial reference frame (ITRF), ITRF2020. And secondly, by carefully describing these sessions in terms of processing as well as results compared to other legacy S/X sessions, we hope to provide the IVS observing program committee with enough information to consider including the upgraded Australian stations in their routine legacy S/X observations.

Mixed-mode observations have also been performed by other groups within the IVS, mainly for the purpose of determining the local tie vectors between existing and new VGOS telescopes. Varenius et al. (2021) describe mixed-mode observations between the legacy and the VGOS twin telescopes at the Onsala Space Observatory. Using X-band phase delay observations, they successfully determine the coordinates of the new twin telescopes and investigate other systematic errors. Similar investigations are underway in Wettzell (*personal communication*). Mixed-mode sessions containing both telescopes at Kokee Park Geophysical Observatory, Hawaii, are described in Niell et al. (2021). Besides dedicated X-band only tie sessions for Kokee Park, their publication also contains a detailed description of session RD1810, when three VGOS antennas were added to a network of six legacy S/X stations. The adopted mixed-mode requires compromises due to the samplers in use at these stations only allowing fixed frequency channels. The observing mode and used procedures were subsequently applied for a few more (RD2005-RD2007) IVS mixed-mode sessions containing more of the new VGOS stations. These IVS mixed-mode sessions predominantly served the purpose of tying the new VGOS stations into the ITRF.

In comparison to the work above, the here presented mixed-mode sessions differ in their mode: the upgraded VGOS stations Hb and Ke solely aim to mimic legacy S/X and the VGOS-VGOS baseline is not correlated in full VGOS mode (i.e. a wideband delay including differential ionospheric estimation). Consequently, we do not expect improved results in terms of accuracy or precision compared to regular legacy S/X observations. We believe, that the Australian mixed-mode observing program has a simplified processing compared to the IVS mixed-mode sessions described above and may help to overcome current reservations over performing more mixed-mode observations due to the extra work that is needed at the correlator. Since the Australian VGOS stations are not new telescopes, they already have an ITRF position and the AUM/AUA sessions aim for continuation of the time series and contribution to global experiments rather than focussing on local ties. As such, these AUM/AUA sessions present a uniquely extensive set of experiments (37 24-h sessions), allowing to investigate their suitability for the standard IVS products through studying the station time series and comparing them to legacy S/X results. The purpose of this work is to encourage the community to include the Australian VGOS stations in standard legacy S/X VLBI. The idea is to allow for the continuation of the station time series. Moreover, it prevents a gap in the current IVS network due to the lack of the Australian stations.

## Technical specifications

Mixed-mode operation, in the context of this paper, is the attempt to operate the new VGOS receivers in a legacy-compatible mode. In this section, the technical details and signal chains are described, first for the legacy stations (Yg, Ho, Ht, Ww, and Ke in 2018) followed by an introduction to the Australian VGOS system at Hb and Ke (from mid-2019 onwards). It is then discussed how compatibility between the two systems can be achieved, emphasising the peculiarities of the introduced mixed-mode observations compared to routine legacy IVS S/X observations. It shall be noted that the described mode was specifically designed for the Australian array, where we benefit from the flexibility of the digital down converter (DDC) mode in the DBBC3 sampler, allowing arbitrary frequency bands.

### Legacy stations

The legacy telescopes observed using the common S/X receiver chain. The receivers are equipped with low noise amplifiers operating either at ambient temperatures (Yg, Ke and Ww) or using cryogenic cooling for better performance (Ht, Ho). With the exception of the 26m antenna in Hobart, the AUSTRAL array consists of small (12m in Yg, Ke, and Ww, 15m Ht) and moderately fast telescopes, with more details given in Plank et al. (2017) and Lovell et al. (2013). Approximate antenna sensitivities given in *System Equivalent Flux Densities (SEFD in Jansky)* are between 4000 Jy and 5000 Jy in both X-band and S-band for Yg, Ke, and Ww. The larger dish sizes and cooled systems of Ht and Ho give better performance of about 2000 Jy in X-band (1400 Jy in S-band) for Ht and 1400 Jy (1600 Jy in S-band) for Ho. For legacy S/X operations, the signal is down-converted against a fixed local oscillator via a mixer at the receiver and transported via coaxial cables to the control room and samplers. The stations are equipped with DBBC2 digital samplers and Mark5 or FlexBuff (Lindquist and Szomuru 2014) recording systems, allowing a typical data rate of 1 Gigabits per second (Gbps). In terms of operations, the data from Hobart, Ht and Ww are typically e-transferred using the program jive5ab^3^ to either Hobart or Vienna for correlation. In the case of Yg and Ke, a lack of fast internet connections does require physical shipping, either of the Mark5 modules or, more recently, of the FlexBuff disks directly.

### Australian VGOS upgrade

While the AuScope VLBI array had already originally been designed for VGOS, the actual receiver upgrade began in 2015. Starting in Hobart, a prototype wideband feed was installed, tested and improved, with the final version of the feed installed in mid-2017. The wideband receiver was designed and built by Callisto, using Stirling cycle cooling. It is equipped with the QRFH feed (Akgiray et al. 2013), sampling signals in two linear polarisations (hereafter referred to as X and Y).

**Fig. 2:**
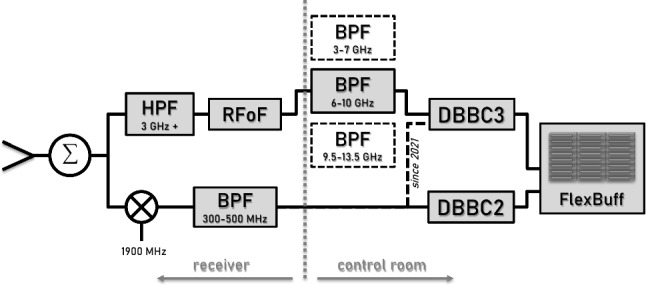
Signal chain installed at Hobart and Katherine. While the VGOS signals are sent through a high-pass filter (HPF) and are transported at sky frequencies via a fibre connection (RFoF) to the control room, signals below 3 GHz are down-mixed and sent via the old coax cable to the control room. The setup shown is identical for both polarisations

The new VGOS system, which is planned to be installed at all three sites, has evolved to a three frequency band system using a DBBC3 and FlexBuff for the backend. As illustrated in Figure 2, signals above 3 GHz are sent through a high-pass filter and then via the radio frequency (RF) over fibre (RFoF) link to the control room. The signals below 3 GHz are mixed with a 1900 MHz local oscillator signal and sent via the old coax connection.

In the control room, the VGOS signal is split and, using 4 GHz wide RF filters, can be input into the DBBC3 (1 input per polarisation). The three overlapping 4 GHz wide bands are at 3-7 GHz, 6-10 GHz, and 9.5-13.5 GHz. The DBBC3s have 6 inputs which are used for three frequency bands at dual polarisation. Using the DDC mode, this allows for full VGOS compatibility. However, in the current DDC_V (V123) configuration, bandwidths are fixed to 32 MHz per channel and 8  channels per intermediate frequency (IF).

As shown in Fig. 2, when installing the new VGOS chain, frequencies below 3 GHz (including the legacy S-band) were not fully excised, but remain accessible in the control room through the existing coax cable. With the feeds nominal operating range of 2.3-14 GHz, there is sufficient sensitivity to detect sources in S-band. The signal can then be digitised either with the old DBBC2 or, more recently, also through the DBBC3 and subsequently recorded with the FlexBuff.

### Mixed-mode observations

The mixed-mode observations presented here incorporate a standard legacy S/X observing mode, namely the 1 Gbps AUSTRAL mode (Plank et al. 2017), with 16x16 MHz channels and S-band restricted to 2.2-2.3 GHz^4^. The X-band channels are placed at (lower channel edge) 8212.99, 8252.99, 8352.99, 8512.99, 8732.99, 8852.99, 8892.99, and 8932.99 MHz, with the first and last channel being recorded in both upper and lower sideband. Six channels are placed contiguously in S-band at 2200.99, 2216.99, 2232.99, 2248.99, 2264.99, and 2280.99 MHz. S-band differs from other IVS experiments (such as R1, R4 sessions), in order to avoid strong local radio frequency interference (RFI) at Hobart above $\approx 2.3~\hbox {GHz}$. For the mixed-mode observations, the aim is for the VGOS receivers to be operated as legacy stations. So how compatible are those two systems?

- **Frequency coverage**
  The nominal operating range of the QRFH feed is $\approx 2.3$–$14~\hbox {GHz}$. Despite the problem of severe RFI in Hobart - which make local Ho-Hb tests impossible - sensitivity in S-band appears reasonable^5^. The legacy X-band is covered by the 6-10 GHz filter and fringes on the Ho-Hb baseline are easily obtained without special conditions being applied in the scheduling. For recording X-band, one only needs two (for dual-polarisation) inputs for the DBBC3. If one wants to use and combine both S- and X-frequencies, one has to account for the intrinsically different delays due to two different signal paths between the receiving feed and the FlexBuff recorder.
- **Polarisation**
  While in legacy S/X VLBI typically the right-hand-circularly polarised signal (RCP) is used, the VGOS signal comes in two linear polarisations (X, Y). Hence, one needs to handle the cross-polarisation products to get full sensitivity or accept a $\sqrt{2}$ loss in sensitivity.
- **Recording**
  The DBBC3s are used in DDC mode (using firmware V123), which has a fixed channel bandwidth of 32 MHz. This means, that for the chosen observing mode with 16 MHz channels, additional data is recorded which is later on discarded in the correlation step. However, with the benefit of a contiguous frequency coverage in S-band, one can again avoid wastage through careful channel selection.

With the oversampling (32 MHz channels instead of 16 MHz) in X-band, the total data rate that needs to be recorded is 3 Gbps, compared to the 1 Gbps for the legacy system. This number does not change when the DBBC3 is also used for sampling the S-band data, since the contiguous channel distribution in S-band allows to pack two channels into a single 32 MHz DBBC channel.

The data was recorded onto a FlexBuff system. The removable drives of this system are directly shipped and subsequently inserted into another compatible machine in Hobart for correlation.

For the majority of the mixed-mode experiments, the VEX (VLBI Experiment Definition)^6^ entries detailing the recording mode information for the DBBC3 and the FlexBuff for the VGOS stations (Hb, Ke) were created by hand. The observations themselves were steered through the NASA Field System^7^ (FS) plus some additional scripts to configure the backends. Full incorporation into the FS and automation of the currently necessary additional scripts has only recently been implemented.

Once theoretical compatibility was confirmed, first test observations were done in May 2018 (McCallum and McCallum 2019). While experiencing some technical problems with the observations, the technique itself was found to be viable and it was decided to organise a large-scale mixed-mode observing program of multiple 24-h sessions.

## Mixed-mode sessions

A significant benefit of the AuScope VLBI array is its priority availability for geodesy and, as it is managed by a single institution, additional short notice observations can be easily performed. A first set of ten 24-h sessions (AUM01-10) was observed in 2018, following the initial tests described in McCallum and McCallum (2019). While this set of observations was quite successful, a detailed analysis was deferred due to other commitments at that time.

In 2020, the monthly AUA sessions organised in collaboration with the Vienna University of Technology (VIEN) adopted the mixed-mode technique. Besides the three AuScope telescopes (Hb, Ke, Yg), Ho, Ww, and Ht are scheduled in these sessions when available. While these sessions are ongoing, for this paper data from 15 AUA sessions (AUA060-AUA074) was used.

The AUM program was resumed in July 2020, with another 17 mixed-mode sessions so far (AUM017-AUM033). This new set of AUMs routinely observed with a four-station network (Hb, Ke, Yg, Ww), with a fifth station (Ht) added since April 2021. The different session-codes (AUM or AUA) have historical reason (mainly who was scheduling and correlating these sessions) and also previously had a slightly different station network. As for the technique itself, both AUA and AUM sessions are identical. In this work, data from 37 successful 24-h sessions was used.

Table 1 gives an overview of all mixed-mode sessions used in this study. As shown in column four, the observations were either scheduled in Vienna or at UTAS, with the responsible person at Vienna moving to ETH Zurich in 2020 using the DACH affiliation from then on. Similarly, the correlation was also shared between UTAS and VIEN, with UTAS being responsible for all sessions since 2021, while VIEN took more responsibility for IVS VGOS correlation. The number of scheduled observations (column 6) gives some insight into improving schedule generations, e.g. when antenna SEFDs were adapted to the actual performance or other scheduling parameters were changed. It is also worth pointing out that the AUA programme in 2020 included the *Southern Intensive* sessions (Böhm et al. 2022), 1-h *intensive* sessions comprising three stations with the aim to determine dUT1. Accounting for time to pause and load the different observing files, the AUA sessions in 2020 include one block of down-time for Ht, Yg, and Hb of 80-minutes duration.

### Scheduling

For the scheduling, the VieSched++ software (Schartner and Böhm 2019) was used. For the time being, the AUM/AUA sessions are scheduled in a pure legacy mode. This means that for the a priori signal-to-noise-ratio (SNR) calculation (see Eq. 1), which is used to determine the scan length for each observation, we assume the legacy SEFD of the receiving telescopes and calculate the SNRs without accounting for the mixed and quasi-Stokes-I baselines between the stations with the upgraded VGOS receivers. As discussed in more details below (Sect. 4.2), correctly accounting for the slightly different observing modes for the three possible baselines (legacy-legacy, legacy-VGOS station, VGOS-VGOS) is not trivial. However, since the differences are small (and the scanlength calculations and SNR targets are chosen with a generous safety margin allowing for errors), for the time being the pure legacy approach in scheduling was found to be sufficient. As a general rule, the AUM sessions have about 20 scans per station per hour. This number is rather low (e.g. compared to previous AUSTRAL sessions, Plank et al. (2017)), since the antenna sensitivities were assumed rather conservatively and station performance of Yg is currently below nominal levels. With regard to the actual (legacy) SEFD for the new VGOS systems, we found that they are slightly worse (between 6000 and 8000 Jy) than the legacy receiver performance (4000-5000 Jy) in both S- and X-band. These values are also very sensitive to the performance of the DBBC3, whose reliability and performance are still being optimised.

The generation of a VLBI schedule can be seen as a challenging optimisation problem. Various different optimisation criteria exist such as station sky-coverage, number of observations, scan duration, and station idle time. Some of these criteria are in conflict with each other, e.g. the need of having a good station sky-coverage over short time intervals for precise determination of tropospheric delays, which emphasises long slews between scans, and the requirement to derive a high total number of scans and thus observations for a good redundancy during analysis, which emphasises short slew times to maximise observing time. Therefore, the goal is to find a good balance between these optimisation criteria (and other scheduling parameters) to obtain a high-quality schedule.

Since July 2020 (session AUA066), the generation of the AUA schedules is fully automated. For every session, $\approx 2000$ different schedules are iteratively generated (Schartner and Böhm 2020). These schedules differ w.r.t. the station weights used during scheduling and the relative importance of scheduling optimisation criteria mentioned before.

The optimisation of the scheduling parameters is performed based on an evolutionary strategy proposed in Schartner et al. (2021). First, a total of 300 randomly sampled scheduling parameter values (e.g. station weights and relative importance of different optimisation criteria) are selected and the corresponding schedules are generated. Based on Monte Carlo simulations, the expected precision of the estimated geodetic parameters is assessed and the best performing scheduling parameters are selected—the so-called parents.

In the second iteration, the parents are used to generate a total of 120 offspring, the next generation of improved scheduling parameter values. This is done by performing cross-over and mutation of the parent parameter values. Next, the corresponding schedules of the offspring-parameters are generated and simulated to assess their expected precision of the estimated geodetic parameters and the next set of parents is selected. These steps are repeated fifteen times to ensure that the scheduling parameter values have enough time to converge towards a minimum. Finally, the best performing schedule is selected and distributed to the stations.

In column 7 of Table 1 the number of successful individual observations that are actually used in the analysis is given as a percent of the number of scheduled observations. While percentages between 80 or 90 are typical for IVS experiments, anything below that can only be explained by a more serious issue at one or more stations. Some of these issues are explained in the last column of Table 1. It is obvious that for multiple AUM/AUA sessions there were significant problems. One reason for that is the slightly more complicated setup for the mixed observing mode. The new backends and recording instruments at the VGOS stations sometimes caused difficulties and errors from observers used to working only with legacy S/X observations. Another reason was the novelty of the VGOS instrumentation itself, with receivers and backends still in debugging and trial mode during parts of this mixed-mode campaign. Five sessions (marked in red in Table 1) have been identified as yielding simply too little or bad data and are excluded from the subsequent analysis. While many other sessions also may have various significant problems, we believe that the overall sample is large enough to prove the suitability of the AUM mode for geodetic observations. In particular, the good performance of the latest sessions provides optimism for more reliable performance going forward. The mixed-mode program is ongoing, with one session of each type (AUM and AUA) occurring once per month at the moment.

**Table 1:**
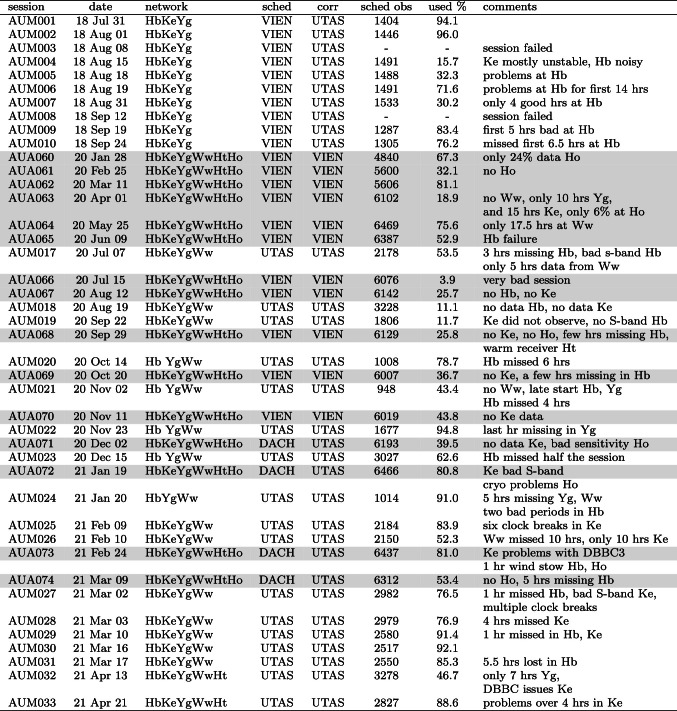
Mixed-mode session schedule. All sessions were of 24-h duration. The session code, date and observing network are given, along with the information about the responsible institution for scheduling and correlation. In the sixth and seventh columns the number of scheduled observations and the percentage of actually used observations are shown. AUA sessions are marked with a grey background. Sessions highlighted in red were excluded from the analysis

## Correlation and post-processing

After the observation, the data of all stations has to be transferred to a single location for correlation. For most of the sessions, the local correlation cluster at the Mt. Pleasant Observatory in Hobart was used. The correlation was performed in one pass, using zoom bands for the VGOS data with only the 16 MHz *legacy* channels being retained in the output. The correlation software DiFX (V 2.6.2) (Deller et al. 2007, 2011) was used, processing multiple datastream files per recording. The Vienna Scientific Cluster (VSC-4) with 480 parallel cores was utilised for correlation and fringe fitting in Vienna.

**Table 2:** Options in Fourfit during the fringe fitting step. There are three different baseline combinations in the AUM/AUA sessions, which are all treated with specific settings and produce marginally different products. In Fourfit processing, the VGOS stations have their X and Y polarisations labelled as R and L

baseline combination	products	Fourfit option	Fourfit output
legacy-legacy	RCP-RCP	-PRR	RCP fringe
legacy-VGOS	RCP-X, RCP-Y	-PRR+RL	“quasi-RCP fringe”
VGOS-VGOS	XX, YY, XY, YX	-PI	quasi Stokes-I solution, nominally $\sqrt{2}$ better sensitivity

The subsequent fringe fitting was done in Fourfit (part of the HOPS package, MIT/Haystack 2021), processed separately at S- and X-band. For all data, the manual phasecal option was applied. This was due to the fact that initially the phasecal system in Hobart was inoperable, but moreover because a multi-tone phase calibration on 16 MHz channels proved difficult due to the 10 MHz spacing between individual phasecal tones. A legacy station (usually Yg) was chosen as the reference station throughout. It should be emphasised that the same DiFX and Fourfit versions were used, and the same strategy for processing the raw data was applied to ensure consistency between both correlators.

At the VGOS stations, we require additional processing steps. Prior to establishing the manual phase offsets, one has to solve for a single band delay offset per sampler. Here, the delay of each polarisation of S- and X-band is determined separately, for a total of four terms. The station-based additive phase offsets per channel are then obtained from a scan on a baseline to the reference station.

Depending on the baseline, a different number of fringe fitting products are produced (see Table 2): on a legacy baseline, one gets a single right-hand-polarisation RCP-RCP product. On a legacy-VGOS baseline, there are two products, RCP-vertical(Y) and RCP-horizontal(X). Finally, on a VGOS baseline there are the full four products (XX, XY, YX, YY) determined. The effectiveness of a manual phasecal solution is checked both for the calibrator scan and then throughout the session for stability.

In terms of operating the Fourfit program, the -P option (enabling polarisation selection) was used. Specifically, -PRR for the legacy-legacy baseline, -PRR+RL or -PRR+LR (dependent on baseline) on the legacy-VGOS baseline and -PI on the VGOS-VGOS baseline. The latter is the full quasi-Stokes-I solution, taking the differential parallactic angle into account. While on the mixed baseline the nominal RCP-RCP sensitivity was expected, the full Stokes-I solution should theoretically give improved sensitivity by $\sqrt{2}$ compared to the RCP-RCP solution.

It should be noted here that a theoretical investigation into the effects of polarisation leakage was not part of this study. As addressed in Corey (2007), the effects of leakage (expressed as D-terms) remain present as first order effects rather than as second order (as in LCP-leakage into RCP receivers) and thus may contribute to systematic errors larger than the few ps-scale reported by Bertarini et al. (2011). The analysis presented in the next section is aimed at detecting these effects, and any other potential errors at the analysis level but a cursory examination of closure delays shows no significant offsets or increased noise when comparing, for example, the Ho-Ww-Yg triangle with Hb-Ww-Yg. A thorough handling of the polarisation leakage in VGOS is still topic of current investigations^8^.

The results of Fourfit are then compiled into a vgosDB database for the geodetic analysis.

### Data volumes and automation

An important step and often the limiting factor in VLBI operations is the data transport of the raw data recorded at each station to the correlator. For full VGOS, the expected data volumes are tens of TB of raw data per station per observing day, which subsequently has to be transferred to and processed at the correlator. As of today, with VGOS operating at the level of one 24-h session per fortnight, already these data volumes pose problems for the existing infrastructure. Storing the raw data until the processing has finished quickly builds up, especially when there are delays in the transport or processing.

For the Auscope VLBI project, these mixed-mode sessions serve as testbed for handling large data volumes and hopefully help towards better preparedness for full VGOS operations. A complete 5-station AUM session has about 60 TB of data. This splits into about 7 TB for Yg and a bit less ($\sim 4$-5 TB) for Ht and Ww which have fewer observations due to their location on the edges of the network. The largest contributions come from the VGOS stations in Hb and Ke ($\sim 20~\hbox {TB}$ each), which need to record three times more data due to the 32 MHz band limitation of the DBBC3 at the time. Data from Ht and Ww are e-transferred, typically within 24-h after the experiment. Yg data are physically shipped, though with regular R1/R4 sessions this transport is well organised and normally arrives in Hobart within about 1 week. The current bottleneck is Ke, where the data are shipped on FlexBuff disks. As a remote site, transfers are initiated remotely and have been prone to interruption and errors leading to lost time and duplication of effort. This has been reduced through regular practice.

Once all data are available at the right place in Hobart, the correlation proceeds taking 1-2 days. Without any major problems, post-processing takes less than 1 day until the final vgosDB can be released.

Overall one can say that the AUM/AUA sessions with a fortnightly cadence have pushed our data storage logistics and triggered procedural and technical improvements. Currently we have about 600 TB of storage available in Hobart and are using a 200 core cluster for correlation.

These regular observations further triggered improvements in automation. As mentioned above (Sect. 3.1), the scheduling of the AUA sessions is fully automated. The next step is to streamline and minimise human interaction in the post-processing. Due to the current scheduling practice for mixed-mode sessions, which assumes the legacy S/X configuration, modifications of the correlation VEX file are essential but routine to represent the recorded data of the VGOS stations. Including the polarisation combination process at the fringe-fitting stage and the delay and phase offset determination for manual phasecal, all steps are automated by the dynamic observing program (Dynob) for the AUM sessions from the start of 2021. We also use a custom m5time script, rather than the vsum program, for the generation of filelists for correlation. For a typical AUM session, this reduces the time needed for this step from about 2 hours to under 2 minutes.

### Sensitivity analysis

In this section we investigate the different sensitivities on the three baseline combinations (legacy-legacy, mixed, VGOS-VGOS).

**Fig. 3:**
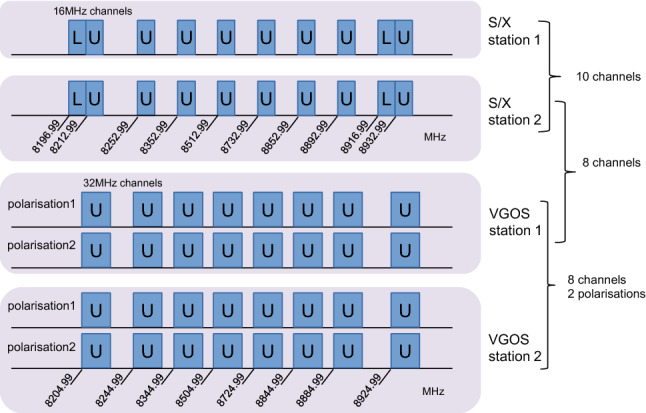
Number of IF channels in X-band used in the AUM/AUA experiments. We illustrate the datastreams from the legacy S/X stations and the stations that have been upgraded with VGOS equipment. The individual channels are illustrated, with the corresponding frequencies given in MHz representing the lower edge of each channel. In legacy S/X VLBI observations, mostly the upper side band (U) is used, with typically two channels also observed in lower side band (L). The AUM/AUA observing mode uses 16 MHz channel width, though 32 MHz channels and two polarisations are recorded at the VGOS stations. When pairing different baselines, this leads to a different amount of common channels, as illustrated on the right

In Figure 3 we illustrate the recorded channels in X-band, for the legacy stations and the VGOS stations. At the legacy stations, the recorded channels match the chosen mode, recording eight upper sideband channels (U) and two lower sideband channels (L) with a channel width of 16 MHz. At the VGOS sites, the current backends require us to record 32 MHz wide channels (only 16 MHz are correlated via zoom-bands), which are placed in a manner that the intended 16 MHz channel lie in the middle of the 32 MHz wide recordings. The data are recorded in two polarisations, and there are no lower sideband recordings available at the VGOS sites, as DiFX correlates all zoom-bands as upper sideband channels.

For each observation, depending on its station combination, there is a different amount of common datastreams available for correlation: in X-band, we have 10 channels for legacy-legacy, 8 common channels for legacy-VGOS, and 8 channels but two polarisations for VGOS-VGOS baselines. For S-band, legacy-legacy and mixed observations both have 6 common channels, with the second polarisation doubling the channels to 12 for VGOS-VGOS baselines.

In geodetic VLBI, the achieved signal-to-noise ratio (SNR) of an observation is important for two reasons. Firstly, the theoretical precision of the derived broadband delay is directly proportional to the achieved SNR, meaning that higher SNR gives better precision. Secondly, in order to get a good balance between a high number of observations within a session and good precision of an individual observation, a target SNR is set in the scheduling process. Typically an SNR value of 20 (15 in S-band) is used during scheduling, determining the scan length of each observation reaching this value. The SNR can be calculated following Eqn. 1, taking into account the source strength *F* in Jansky, the antenna sensitivities of the participating two stations measured in *SEFD*, the scan length *T* in seconds, as well as the number of channels $N_{ch}$ and the channel bandwidth *B*.

1 $$\begin{aligned} SNR=\frac{F}{\sqrt{SEFD_{1} \times SEFD_{2}}}\frac{\sqrt{2\times B \times N_{ch} \times T}}{1.75} \end{aligned}$$

Eqn. 1 is valid for RCP (or LCP) data, already accounting for a $\sqrt{2}$ loss in SNR due to the single polarisation.

In order to compare the different SNR on the various baselines, we define SNR factors. Defining a legacy-legacy observation as the standard case, one can then calculate these SNR factors for the two other configurations.

Considering the different number of channels and Eqn. 1, one finds a factor of $\sqrt{8/10}=0.89$ for the mixed case in X-band and no change in S-band. For the VGOS-VGOS observations, one has only eight frequency channels available, but a second polarisation. Thus we find $\sqrt{8/10}\times \sqrt{2}=1.27$ for X-band and $\sqrt{2}=1.41$ for S-band.

**Fig. 4:**
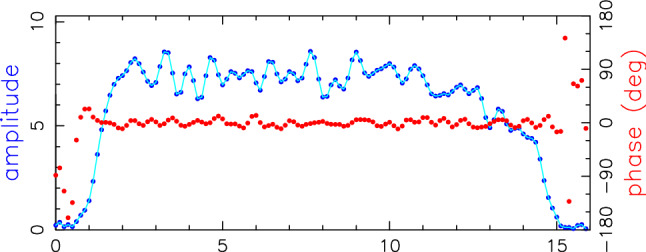
Averaged power spectrum in X-band for a mixed-mode observation on the Ke-Yg baseline. The figure is taken from the Fourfit output file, which is the result of the fringe fitting. The blue curve represents the mean bandpass shape, averaged over the scanlength and all individual channels. One finds significantly lower power at the edges of the channel. Note that this example is of a mixed baseline, with only one DBBC2 causing the drop at the edges. This effect may be even larger on a legacy–legacy baseline

Besides the varying number of channels, there is another effect that impacts the achieved SNR. When recording a 16 MHz channel with the legacy backend, the bandpass filter in the backend typically falls off towards the edges of the band. This is illustrated in Figure 4. When studying the power of the correlated signal over the 16 MHz channel, one finds that one loses about 2 MHz on each side. Hence, the effective bandwidth is more like 12 MHz instead of the envisaged 16 MHz.

**Fig. 5:**
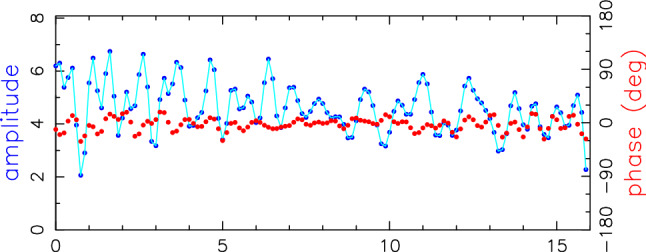
Averaged power spectrum in X-band for a VGOS observation on the Hb-Ke baseline. The figure is taken from the Fourfit output file, which is the result of the fringe fitting. The blue curve represents the mean bandpass shape, averaged over the scanlength and all individual channels. One finds constant power over the full 16 MHz channel

For the VGOS backends, this is different. Since we are recording 32 MHz wide channels, with the desired frequency range placed in the middle, there is no detrimental bandpass effect on the edges, instead we find constant power across the full 16 MHz channel (Fig. 5).

Following the theoretical relations from above (Eqn. 1) and using the VGOS-VGOS full 16 MHz case as the optimal, one finds SNR factors of $\sqrt{12/16}=0.87$ for the legacy and mixed observations in X-band due to the bandpass effect. For S-band (and the case of recording the data with the DBBC3), the situation is slightly different. Since two frequency channels are packed into a single recorded channel, one does see a bandpass effect on one side of each frequency channel leading to effective bandwidths of 14 MHz. These correspond to a factor of $\sqrt{14/16}=0.94$ in S-band. One shall bear in mind that this is only an estimation based on visual inspection of the data. Furthermore, it should be noted that the reported SNR from Fourfit takes the signal chain into account in scaling the theoretical delay error accordingly.

A summary of the theoretical SNR factors is shown in Table 3.

In total, combining effects due to the varying number of common channels and the bandpass, we get SNR factors of about 1.5 for both X-band and S-band for the VGOS-VGOS baseline of Hb-Ke and expect it to be more sensitive when compared to the legacy-legacy case. These values closely match the achieved SNRs of the actual observations and should be used for scheduling in the future.

## Results

After the fringe fitting, the vgosDB databases are progressed from level 1 through level 3, at which stage the data is processed with $\nu$Solve (Bolotin et al. 2012). Here, the ambiguities are resolved and the S- and X-band data is merged to the ionosphere-free linear combination. The results are version 4 databases, in vgosDB format. The data is then made publicly available via the IVS^9^, currently submitted and cross-checked by the GSFC analysis group.

In terms of results, the AUM/AUA sessions are investigated for the estimated station positions. The intention is to show the suitability of the data for the continuation of the station time series as well as a comparison of these regional sessions’ results with global (standard legacy S/X) sessions.

### Analysis of sessions

For the geodetic analysis, the VieVS environment (Böhm 2018) is used. Each session is analysed individually with standard settings for clocks (piece-wise linear offsets (pwlo) with time interval one hour, one rate and one quadratic term relative to a reference clock) and atmospheric delay parameters (zenith wet delay as pwlo every 30 minutes with relative constraints 15 mm and tropospheric gradients as pwlo every 3 hours with relative constraints 5 mm). Additionally, significant baseline-dependent clock offsets are estimated according to Krásná et al. (2021) to account for baseline-dependent offset in the post-fit residuals. In general, the modelling of the group delays is carried out following the Conventions of the International Earth Rotation and Reference Systems Service (IERS) (Petit and Luzum 2010) with online updates.

**Table 3:** Theoretical SNR factors for different baseline combinations. We account for the effects of different number of channels as well as the bandpass shape. In the last column, both effects are combined and rounded total values are given

Mode	Channels		Bandpass		Total	
	X	S	X	S	X	S
legacy-legacy	1.00	1.00	0.87	0.87	1.0	1.0
legacy-VGOS	0.89	1.00	0.87	0.87	0.9	1.0
VGOS-VGOS	1.27	1.41	1.00	0.94	1.5	1.5

When estimating station coordinates, one typically uses no-net-rotation (NNR) and no-net-translation (NNT) conditions to fit the session results into the corresponding reference frame. In such a case the a priori reference frame is considered free of errors and the linear condition equations allow the adjustment of a free geodetic network. For the small and regional network of the AUM/AUA sessions, the choice of the underlying frame and the datum is even more important. The sessions are also not well suited to reliably estimate Earth orientation parameters, moreover, the decision to do so or not can influence the estimated coordinates significantly. In order to allow comparison with global data and gathering information about an individual station position time series, the analysis strategies are varied as described below.

#### Reference solution Vie211015

**Fig. 6:**
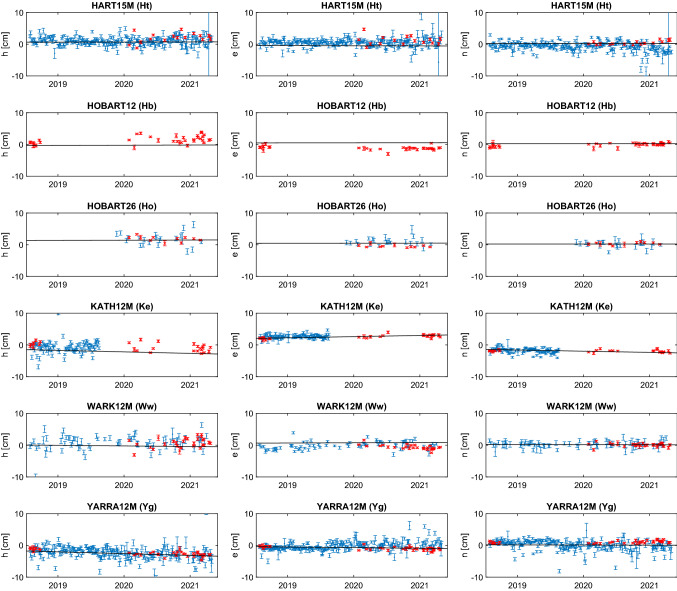
Station times series in height (h), east (e) and north (n) components for the AUM/AUA sessions (red) and R1/R4 sessions (blue) in the same time period. Offsets and formal errors are given in cm, with respect to ITRF2014. The black lines are corrections to the ITRF2014 as estimated in Vie211015. Hb has not participated in R1/R4 sessions since 2017

The currently latest realisation of the international terrestrial reference system (ITRS) is ITRF2014 (Altamimi et al. 2016), which is the underlying frame of our calculations. Since only about three years of data from the Australian stations were included in ITRF2014, the upcoming new solution with more than ten years of data (ITRF2020 in preparation) is expected to show large offsets in the estimated velocities. Since ITRF2020 has not been published yet we use Vie211015^10^ for comparison. It is a global solution generated with the least squares adjustment of the recommended 24-h VLBI sessions^11^ until the end of 2020 for the upcoming ITRF2020 release. From the sessions mentioned in this publication, the R1/R4 and AUA sessions are included, but the AUM sessions are not used. The NNT/NNR conditions are applied on selected 22 stations w.r.t. ITRF2014. From the stations participating in the AUM/AUA sessions only Ho is included in the datum. Source coordinates are determined in this global solution as offsets applying NNR on all ICRF3 defining sources (with exception of 0700-465, 0742-562, 0809-493), and Earth orientation parameters are estimated on a session-wise basis. The difference between Vie211015 w.r.t. ITRF2014 is depicted as black line in Figure 6.

#### AUM/AUA global solution

For comparison, a global solution including the regional AUM and AUA sessions only is computed. The station positions are estimated as offsets to ITRF2014 coordinates including all stations in the NNT/NNR condition while velocities are kept fixed. Earth orientation parameters are estimated as session-wise offsets to the IERS 14 C04 series (Bizouard et al. 2019) and source positions are fixed to ICRF3. As shown in the next sections, the corrections to the ITRF2014 coordinates (Table 4) from this solution are distorted by applying the usual parametrisation strategy, i.e. all stations in the NNT/NNR condition w.r.t. underlying TRF and estimation of the Earth orientation parameter (EOP) offsets. The inclusion of all stations in the NNT/NNR condition is problematic since velocities of the Australian sites have large formal errors in ITRF2014. This is because of the limited observation history of three years which results in wrong a priori position of the stations during the AUM/AUA sessions. This is true especially for Ke and Yg, since the velocity for Hb was tied to Ho with long measurement history. Concerning the EOP, estimation of all five parameters in a regional network distort the station positions at edges of the network, in case of the AUM/AUA sessions it applies particularly to Ht and Ww.

**Table 4:** Coordinate offsets and formal errors of the AUM/AUA stations with respect to ITRF2014 as estimated in a global solution of all mixed-mode sessions as described in Sect. 5.1.2. Values are given in cm

Station	dx	dy	dz	dh	de	dn
HART15M (Ht)	$0.88 \pm .10$	$0.35 \pm .04$	$-0.1 \pm .02$	$0.89 \pm .07$	$-0.10 \pm .05$	$0.32 \pm .03$
HOBART12 (Hb)	$-0.39 \pm .06$	$1.04 \pm .04$	$-0.56 \pm .06$	$1.03 \pm .06$	$-0.67 \pm .05$	$0.19 \pm .06$
HOBART26 (Ho)	$-0.81 \pm .10$	$0.68 \pm .06$	$-0.32 \pm .10$	$0.99 \pm .09$	$-0.14 \pm .07$	$0.48 \pm .09$
KATH12M (Ke)	$-0.98 \pm .06$	$-1.34 \pm .05$	$-1.93 \pm .03$	$0.15 \pm .05$	$1.63 \pm .06$	$-1.95 \pm .03$
WARK12M (Ww)	$-0.14 \pm .09$	$0.11 \pm .04$	$1.58 \pm .06$	$-0.82 \pm .08$	$-0.10 \pm .04$	$1.36 \pm .07$
YARRA12M (Yg)	$1.44 \pm .04$	$-0.84 \pm .06$	$1.23 \pm .04$	$-1.80 \pm .05$	$-0.94 \pm .04$	$0.41 \pm .04$

#### AUM/AUA station position time series

Thus, for the aforementioned reasons, we decided to compute the time series of the station position from the AUM/AUA sessions applying a slightly unusual parametrisation strategy. The time series for each station is obtained from a so-called backward solution run after an individually adjusted global solution of the AUM and AUA sessions. The difference between these several global adjustments is that the particular station of interest is excluded from the NNT/NNR condition w.r.t. ITRF2014. In order to obtain time series of this particular station, its position is reduced from the normal equation system and estimated in a backward solution without additional conditions. The Earth orientation parameters are fixed to IERS 14 C04 time series (Bizouard et al. 2019) and the celestial reference frame to ICRF3. The residual values to the a priori ITRF2014 values in up, east, and north direction s are presented in Figure 6 in red colour.

#### Legacy R1/R4 session-wise solution

Whenever available, results from global IVS R1/R4 sessions^12^ are shown for comparison.

The R1/R4 are adjusted session-wise in individual solution series for each station separately, where the station of interest is always excluded from the NNT/NNR condition w.r.t. ITRF2014. Furthermore, we exclude station Sejong^13^ from the NNT/NNR condition in all solutions since it joined the IVS R1 program at the end of September 2014 and therefore only three months of data are included in the ITRF2014. The reason why we do not estimate the station position time series from a backward solution (as it is the case in the AUM/AUA sessions adjustment) is that the R1/R4 sessions do have enough (more than three) globally distributed stations with good a priori coordinates in ITRF2014 suitable for the NNT/NNR condition. The Earth orientation parameters are estimated as offsets to the IERS 14 C04 series. The positions of radio sources are fixed to ICRF3 with the exception of source 3C48 (0134+329) which exhibits a difference of about -57 mas in declination to ICRF3 (global VLBI solution Vie211015 of the TU Wien group^14^). Therefore, we estimate the position of 3C48 in each session. The time series of the estimated station position offsets to the a priori ITRF2014 are depicted in blue colour in Figure 6.

**Table 5:** WRMS of the station position estimates from the AUM/AUA sessions and R1/R4 sessions (plotted in Fig. 6) w.r.t. Vie211015. Values are given in cm

station	AUM/AUA			R1/R4		
	h	e	n	h	e	n
Ht	1.63	1.33	0.53	1.17	1.02	1.26
Hb	1.05	0.39	0.42			
Ho	0.72	0.23	0.37	1.63	0.88	1.00
Ke	0.96	0.35	0.38	1.25	0.51	0.68
Ww	1.46	0.68	0.49	1.93	1.07	0.87
Yg	0.62	0.48	0.40	1.33	1.05	1.29

### Discussion of results

As Figure 6 depicts, for Yg, Ke, and Ho we find the results in very good agreement with the R1/R4 sessions. Particularly in Ke, the mixed-mode series seems to continue the trends seen in the global sessions, which of course stopped in mid-2019 when the VGOS receiver was installed. It is also clear that the underlying a priori model (ITRF2014) needs to be improved. The results for Ht and Ww also show good agreement with the global sessions, despite the fact that these stations are at the edges of the network. Geometrically, their coordinates are less robust in these sessions, with additional disadvantages in the scheduling process in terms of common visibilities and sky coverage.

**Fig. 7:**
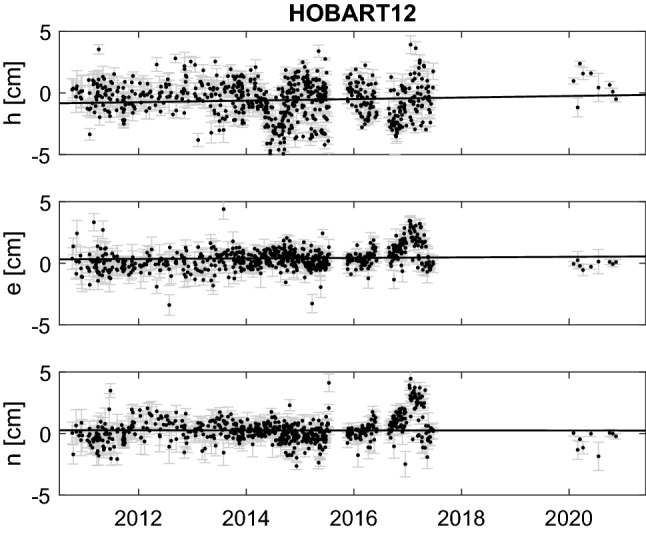
Session-wise estimated height (h), east (e) and north (n) components w.r.t. ITRF2014 for Hb from all sessions included in Vie211015 (i.e. sessions in 2020 are AUA sessions; AUM sessions are not used). The black lines are corrections to the ITRF2014 as estimated in Vie211015

For Hb, these sessions are the first geodetic results since 2017. In Fig. 6 the data shows clear offsets to the a priori positions, of about 2 cm in each the height and the east direction. Yet these offsets are not significant when looking at the full history of Hb data. In Fig. 7 we show session-wise estimated height, east, and north components of Hb from all sessions included in the Vie211015 solution.

As mentioned in Sect. 5.1.1, AUA sessions up to the end of 2020 are included in this solution, while AUM sessions are not. This 10-year timeseries of Hb does show larger systematics or variations of a few cm, and the AUA results seem to continue previous patterns, without showing any new systematic offset. A similar behaviour was observed for the Ke height component, where the discrepancy between the recent data (red and blue marks in Fig. 6) and the reference solution shown (black line) is not significant when studying a longer time series.

A continuation of Hb results is of particular interest since there were always some unexplained effects visible in legacy Hb results: for example, a suspected periodic signal in the height component (see, for example, Fig. 7 in Plank et al. 2017) or larger than expected differences between the local baseline and the site survey (Plank et al. 2015a). There is also a significant discrepancy between the measured (2.1 mm) axis offset at Hobart and the results from estimating the axis offset in a global solution (1.3 cm in 2014, 1.8 cm in 2020).^15^ While the estimated values were used for ITRF2014, since 2020 it is recommended to use the survey values instead. There is strong suspicion that the phasecal signal for the S/X system in the Hb antenna may have been corrupt and might introduce systematic effects in the analysis. A thorough comparison between identical sessions using once the phasecal signal and then applying a manual phasecal solution is currently under investigation.

Next, we looked at baseline lengths and their repeatabilities. While station coordinates are the best way to examine compatibility with the legacy S/X results, baseline lengths remain the ultimate measure of consistency and precision for these mixed-mode sessions. For this comparison we take session-wise solutions with all stations in NNT/NNR condition. The median post fit weighted rms for the AUM and AUA sessions is 40.3 ps which is comparable to the median of the session fit for R1/R4 sessions from this analysis, which is 39.7 ps.

**Fig. 8:**
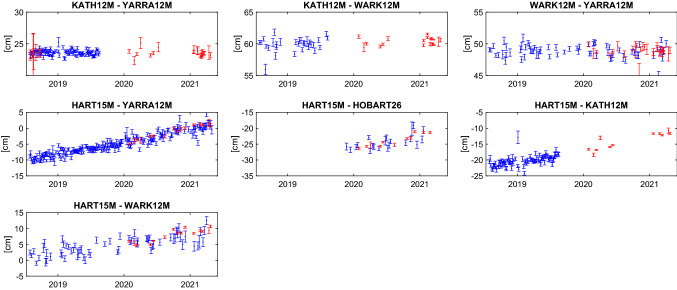
Baseline time series for the AUM/AUA sessions (red) and R1/R4 sessions (blue) in the same time period. Offsets and formal errors are given in cm, with respect to the baseline lengths given in Table 6. Those lengths are rounded to the metre. Only baselines with results for both, AUM/AUA and R1/R4 sessions are shown

In Fig. 8 the baseline length time series are shown for baselines with results for both, AUM/AUA as well as R1/R4 sessions. One again finds good agreement between the legacy S/X and the mixed-mode results.

**Table 6:** Weighted baseline lengths repeatabilities for the AUM/AUA and R1/R4 sessions. Number of sessions used to calculate the BLR is given in brackets. Values were calculated only for baselines that were observed in at least 10 sessions

baseline	length [km]	AUM/AUA (no.)	R1/R4 (no.)
Ke-Yg	2360.367	3.6 mm (28)	3.7 mm (95)
Ho-Ww	2415.319	4.4 mm (10)	–
Hb-Ww	2415.533	4.5 mm (26)	–
Hb-Yg	3211.336	3.7 mm (35)	–
Ho-Yg	3211.457	3.4 mm (11)	–
Hb-Ke	3431.879	5.6 mm (27)	–
Ke-Ww	4752.943	5.5 mm (19)	8.5 mm (30)
Ww-Yg	5362.036	5.2 mm (28)	8.7 mm (68)
Ht-Yg	7848.823	4.2 mm (16)	9.7 mm (213)
Ht-Hb	9167.446	7.3 mm (14)	–
Ht-Ho	9167.666	6.4 mm (11)	15.8 mm (21)
Ht-Ke	9504.495	7.3 mm (11)	9.9 mm (89)
Ht-Ww	10480.989	10.3 mm (15)	19.0 mm (67)

For Table 6, weighted baseline length repeatabilities (BLR) were calculated for baselines with 10 or more common sessions, and compared to the BLR from R1/R4 sessions. When comparing these results from AUM/AUA sessions with those from the R1/R4 sessions, one has to bear in mind that the former has a largely stable station network (and datum stations) while the network in the R1/R4 sessions can vary significantly. This will impact the repeatabilities. As previously pointed out by Plank et al. (2017), this comparison is further distorted by the different observing modes (256/512 Mbps for the R1/R4 sessions and 1 Gbps for the AUM/AUA sessions), which nominally should yield different precision. As visible in Figure 8, the AUM/AUA sessions show smaller formal errors compared to the R1/R4 sessions, which is another explanation for the superior BLR results.

The results in terms of BLR agree well with previous results from legacy S/X AUSTRAL sessions (Plank et al. 2017, Table 5), and are improved for the long baselines to Ht. It is also good to see that BLR from the global R1/R4 sessions have improved for the short and medium baselines, compared to a few years ago.

While the theoretically improved sensitivity on the Hb-Ke baseline between two VGOS stations (see Sect. 4.2 and Table 3) at some point should yield improved results compared to a legacy-legacy or mixed baseline, this effect is not visible yet. We explain this with the fact that these AUM/AUA sessions were mainly conducted during the upgrade process, with multiple sessions experiencing serious problems. In addition, the VGOS stations have not shown their expected performance in terms of SEFD yet.

**Fig. 9:**
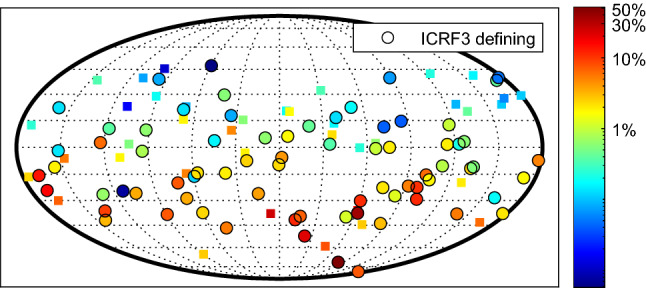
Observed sources in the AUM/AUA sessions. The colours indicate the number of observations, expressed as a percentage of observations of that source in ICRF3. Note the logarithmic scale. ICRF3 defining sources are marked with circles

As a last point, we would like to comment on the sources observed in the AUM/AUA sessions, and their impact on future realisations of the ICRF. As repeatedly stressed by the authors of ICRF3, the latest realisation of the celestial reference frame still suffers under a strong imbalance between northern and southern sources, induced by the comparably low number of available observations in the south. In the available AUM/AUA sessions, a total number of 111 sources were observed, with 73 of them being defining sources in ICRF3. Over all sessions, most sources ($\sim$70) have a few hundred observations, while the other $\sim$40 sources were observed more often, up to a few thousand times. In Figure 9 we show a map of the observed sources.

The visibility of the AUM/AUA network allows to observe sources up to $47^{\circ }$ north on at least one baseline. The colour-coding in Figure 9 intends to show the importance of these mixed-mode sessions for the ICRF: on a logarithmic scale, the colour represents the total number of observations of each individual source in the AUM/AUA sessions, as a percentage of the number of observations of that source used in ICRF3. While the impact for northern sources is rather small ($<1\%$ for the green and blue markers), the importance of the AUM/AUA sessions increases for southern sources, with numbers reaching 5-10%. For 11 sources, AUM/AUA observations would increase the number of observations by more than 10%.

## Conclusions

The Australian AUM/AUA session series provides a large testbed for mixed-mode VLBI observations, combining both classical circularly polarised legacy stations with the new linearly polarised VGOS technology. The collected data is essentially legacy VLBI, though allowing the new VGOS stations to participate. In the AUM project we demonstrate compatibility of the Australian VGOS stations with legacy S/X VLBI and describe the technical details for correlation and post-processing. Most importantly, the analysis of 37 successful 24-h sessions shows good geodetic results for both, the legacy and VGOS stations. Baseline repeatabilities are well in accordance or exceeding the result of previous full legacy S/X AUSTRAL sessions. Estimated station coordinates of the legacy stations are comparable to the results from global legacy S/X sessions and do not show any systematic offsets. For the VGOS stations, these results are currently the only possibility to continue the time series.

For the AuScope VLBI project, these AUM/AUA sessions are important in three aspects, (a) the continuation of the station time series, (b) enabling the operation of AUSTRAL sessions, triggering development in big data handling and increased session cadence and (c) allowing testing and operation of the new VGOS stations. These sessions further offer exciting science, e.g. a deeper investigation of the systematic offset and signals in the Hb time series. While currently it is still suspected that an erroneous phasecal signal may be causing this effect, the fact that the same signal may be present in the mixed-mode data when using manual phase calibration is counter to this hypothesis. Multiple investigations into this effect have not given a clear answer so far, and a dense and long time series would be a new chance to hopefully achieve a resolution. Another topic of research enabled through these observations is to look for systematic errors which might be introduced by using dual linear polarisations at the new VGOS sites. For these reasons, the AUM project will be continued, currently with a cadence of one session per fortnight.

Lastly, the issue of global sessions in mixed-mode shall be discussed here. IVS mixed-mode sessions have been organised in order to tie the new VGOS telescopes into the ITRF. Since they incorporate a slightly different strategy (i.e. also correlating the full VGOS baseline where possible, see Niell et al. (2021) for details), the new Australian VGOS stations have not participated in those sessions. In addition, the processing of those sessions does cause extra work at the correlators and the general enthusiasm for doing more mixed-mode sessions is therefore limited. With this AUM/AUA series, we would like to start the discussion within the IVS to consider including the Australian VGOS stations in standard legacy S/X sessions. While there will be more efforts needed at the processing stage, it will be worthwhile. The roll-out of VGOS is slow and the current cadence of 1 or 2 sessions per fortnight with a limited network will not be able to match the expectations for geodetic products for a while yet. A recent study by Schartner et al. (2020) highlighted the importance of southern-hemisphere stations for the current VGOS network, that only consists of northern-hemisphere stations. Large-scale Monte Carlo simulations revealed that adding a southern-hemisphere station more than halved the formal errors of the earth orientation parameters. In the meantime, more and more legacy telescopes cease their operations, significantly worsening the legacy S/X network and geodetic results. Adding the Australian VGOS stations back in the core legacy S/X observations or regularly observing global mixed-mode sessions would allow to at least maintain the level of legacy S/X results from previous years, until VGOS results will become the new standard. We believe that the addition of mixed-mode sessions is a chance to push the limits of all components of the IVS infrastructure and processing chain. Increased observing days and data volumes will identify insufficiencies in the transport, storage and processing of data, resulting in the necessary improvements required as we move into the VGOS age. Without these significant efforts and developments in big data handling as well as automation in the current processing, the VGOS vision of continuous observations with near real time results will remain unachievable for many years to come.

## Data Availability

All data described in this paper is made publicly available through the IVS, on https://ivscc.gsfc.nasa.gov/products-data/.

## Footnotes

1 https://www.hat-lab.cloud/dbbc3-2/.

2 Yg is observing about 150 IVS sessions per year, Ho about 50. Ho has experienced major structural damage in August 2021 and has not been operational since. In particular the fast-switching geodetic sessions pose troubles for the old Ho telescope. In addition, other commitments in astronomy and spacecraft operations more and more limit the time that Ho will be available for geodesy and the IVS in the future.

3 https://github.com/jive-vlbi/jive5ab.

4 an exemption are sessions AUM001-AUM006, which actually observed at the frequencies standardly used in the IVS R1/R4 sessions.

5 between 6000 Jy and 8000 Jy compared to previously about 4000-5000 Jy as legacy station.

6 https://vlbi.org/vlbi-standards/vex/.

7 https://github.com/nvi-inc/fs.

8 e.g. by using the astronomical PolConvert software (Martí-Vidal et al. 2016).

9 https://ivscc.gsfc.nasa.gov/sessions/.

10 available at: https://vlbi.at/data/analysis/ggrf/trf_vie2020_211015.txt

11 list of ITRF2020 VLBI sessions: https://ivscc.gsfc.nasa.gov/IVS_AC/IVS-AC_ITRF2020.htm.

12 https://ivscc.gsfc.nasa.gov/program/descrip2011.html.

13 https://ivscc.gsfc.nasa.gov/publications/ar2014/nssejong.pdf.

14 available at: https://vlbi.at/data/analysis/ggrf/crf_vie2020_211015.txt.

15 https://raw.githubusercontent.com/anothnagel/antenna-info/master/antenna-info.txt.
